# Wheezing in infancy

**DOI:** 10.1097/WOX.0b013e318216b41f

**Published:** 2011-05-15

**Authors:** Yehia M El-Gamal, Shereen S El-Sayed

**Affiliations:** 1 Pediatric Allergy and Immunology Unit, Children's Hospital, Ain Shams University, Cairo, Egypt

**Keywords:** wheeze, viral infection, bronchiolitis, bronchospasm, infants

## Abstract

Several population-based birth cohort studies documented that 30% of children suffer from wheezing during respiratory infections before their third birthday. Infants are prone to wheeze because of anatomic factors related to the lung and chest wall in addition to immunologic and molecular influences in comparison to older children. Viral infections lead to immunologic derangements that cause wheezing both in immunocompetent and immunodeficient infants. Anatomic causes of wheeze may be extrinsic or intrinsic to the airway. Not every wheeze is indicative of asthma but prediction of asthma in persistent wheezers is possible. Testing for allergy in these infants is worthwhile and can be of significant value in avoidable allergens. Treatment of an infant with wheezing depends on the underlying etiology. Response to bronchodilators is unpredictable and a trial of inhaled steroids may be warranted in a patient who has responded to multiple courses of oral steroids, has moderate to severe wheezing, or a significant history of atopy including food allergy or eczema. Ribavirin administered by aerosol, hyper-immune respiratory syncytial virus immunoglobulin (RSV IVIG), and intramuscular monoclonal antibody to an RSV protein have been used for RSV bronchiolitis in infants with congenital heart disease or chronic lung disease.

## 

Wheezing is a high pitched, whistling sound that occurs when smaller airways are narrowed by presence of either bronchospasm, swelling of mucosal lining, excessive amounts of secretions, or inhaled foreign body. It is heard mostly on expiration as a result of critical airway obstruction [1]. It is polyphonic when there is widespread narrowing of the airways causing various pitches or levels of obstruction to airflow as seen in asthma. Monophonic wheezing refers to a single-pitch sound that is produced in the larger airways during expiration as in distal tracheomalacia or bronchomalacia. When obstruction occurs in the extrathoracic airways during inspiration, the noise is referred to as stridor [2].

Wheezing is common throughout infancy and childhood except in the neonatal period when it is relatively rare. About 19% of 10-year-old children experience wheezing with an average age of onset of 3 years [3]. In addition, several population-based birth cohort studies documented that 30% of children suffer from wheezing during respiratory infections before their third birthday. Recurrent wheezing is common, but most patients outgrow their symptoms by school age [4].

Infant wheezing is sometimes mixed with other causes of noisy breathing including all causes of nasal obstruction in the first 2 years of life. Adenoid hypertrophy is commonly miss-diagnosed as bronchospasm on chest auscultation.

## Pathophysiology

Infants are prone to wheezes because of anatomic factors related to the lung and chest wall in addition to immunologic and molecular influences in comparison to older children [5]. The obstruction to flow is affected by the airway caliber and compliance of the infant's lung. Resistance to airflow through a tube is inversely related to the radius of the tube to the 4th power. In children < 5 years old, small caliber peripheral airways can contribute up to 50% of the total airway resistance. Marginal additional narrowing can cause further flow limitation and a subsequent wheeze [6]. With the very compliant newborn chest wall, the inward pressure produced in expiration subjects the intrathoracic airways to collapse. Flow limitation is further affected in infants by the differences in tracheal cartilage composition and airway smooth muscle tone causing further increase in airway compliance compared with older children. All of these mechanisms combine to make the infant more susceptible to airway collapse, increased resistance, and subsequent wheezing. Many of these conditions are outgrown by the 1st year of life [7].

Immunologic and molecular influences can contribute to the infant's propensity to wheeze. In comparison to older children and adults, infants tend to have higher levels of lymphocytes and neutrophils, rather than mast cells and eosinophils in bronchoalveolar lavage fluid. A variety of inflammatory mediators have also been implicated in the wheezing infant such as histamine and leukotrienes. Fetal and/or early postnatal "programming" in which the structure and function of the lung are affected by factors including fetal nutrition and fetal and neonatal exposure to maternal smoking may also occur [8].

## Etiology

Congenital conditions causing wheezing disorders should not be missed and not all wheezy bronchitis is or will become asthma [1]. Most wheezing in infants is caused by either inflammation or anatomic abnormalities (Table 1).

**Table 1:** Most Common Causes of Wheezing by Mechanism

Diagnostic Category	Cause
Anatomic	Extrinsic to airway
	Lymphadenopathy
	Tumor
	Diaphragmatic hernia
	Vascular ring/aberrant vessel
	Intrinsic to airway
	Tracheomalacia
	Foreign body
	Endobronchial tuberculosis
	Vocal cord dysfunction
	Bronchopulmonary dysplasia
	Congestive heart failure
	Congenital lobar emphysema
Inflammatory/infectious	Asthma
	Bronchiolitis
	Respiratory syncytial virus
	Influenza A and B
	Adenovirus
	Rhinovirus
	Bronchitis
	Pneumonia
	Mycoplasma pneumonia
	Chlamydia pneumonia
	Aspiration pneumonia
	Bronchiectasis
	Alpha 1 antitrypsin deficiency
	Pulmonary hemosiderosis
Genetic/metabolic	Cystic fibrosis
	Immotile cilia syndrome
	Kartagener syndrome
	Metabolic disturbance
	Hypocalcemia
	Hypokalemia

Adapted from Shah (2004) [10].

## Inflammatory disorders

### Infection in previously healthy infants

Bronchiolitis is an acute infectious disease of the lower respiratory tract that occurs primarily in young infants, most often in those aged 2-24 months. Seventy-five percent of cases of bronchiolitis occur in children younger than 1 year, and 95% in children younger than 2 years. Incidence peaks in those aged 2-8 months. Annual incidence is 11.4% in infants younger than 1 year and 6% in those aged 1-2 years. The illness accounts for 4500 deaths and 90,000 hospital admissions per year. Prevalence may be higher in urban areas [9].

Bronchiolitis is usually because of a viral infection of the small airways. Infection of bronchiolar respiratory and ciliated epithelial cells produces increased mucus secretion, cell death, and sloughing, followed by a peribronchiolar lymphocytic infiltrate and submucosal edema. The combination of debris and edema produces critical narrowing and obstruction of small airways. Decreased ventilation of portions of the lung causes ventilation perfusion mismatching, resulting in hypoxia. During the expiratory phase of respiration, further dynamic narrowing of the airway produces disproportionate airflow decrease and resultant air trapping. Work of breathing is increased because of increased end-expiratory lung volume and decreased lung compliance. Recovery of pulmonary epithelial cells occurs after 3-4 days, but cilia do not regenerate for about 2 weeks. The debris is cleared by macrophages [2]. Infection is spread by direct contact with respiratory secretions. Previous infection with the common etiologic viruses does not confer immunity [11].

Not all infected infants develop lower respiratory tract infection (LRTI). Host anatomic and immunologic factors seem to play a significant role in the severity of the clinical syndrome. Infants with preexistent smaller airways and diminished lung function have a more sever course. In addition, respiratory syncytial virus (RSV) infection incites a complex immune response and infants who wheeze express higher levels of interferon-*γ *in the airway and leukotrienes. RSV coinfection with metapneumovirus can be more severe than monoifection [12]. Human metapneumovirus (hMPV), like human RSV, is classified in the Pneumovirinae subfamily of the paramyxoviridae family. However, it is mostly genetically related to avian metapneumovirus. hMPV was first described in 2001 by researchers in the Netherlands as a single negative (stranded RNA) enveloped virus. Two major groups (A and B) and 4 subgroups have been identified to date [13].

There is evidence that deficiencies in antiviral activity and the integrity of the airway epithelial barrier could make individuals with asthma more likely to have severe viral respiratory infections of the lower airway, and thus increase the risk of exacerbation. Advanced molecular diagnostics have identified human rhinoviruses (HRVs) as pathogens frequently causing wheezing illnesses in infants and young children. Wheezing during HRV infection in early life identifies children at particularly high-risk of asthma development [15]. HRV infections promote the expression of factors that have been associated with airway damage and remodeling such as increased production of mediators involved in airway remodeling, including amphiregulin, activin A, and VEGF [16]. It was recently demonstrated that HRV infection of epithelial cells also causes TLR-3 dependent mucin production and up-regulation of epidermal growth factor receptor, a prominent component of epithelial repair [17]. Whether particular strains of HRV are more pathogenic is an open and important question [15].

## Infection in infants with underlying disease

The respiratory tract is the organ system most commonly involved in immunodeficiency disorders. There is often a delay in diagnosis and this delay increases the risk of bronchiectasis and irreversible lung damage occurring before appropriate treatment is given [18]. An immune defect should be considered in any child who has respiratory infections that are unusually severe, recurrent, unresponsive to conventional treatment, or atypical. Common associated features include failure to thrive, which is often secondary to gastrointestinal disease, severe atopic disease such as eczema, and occasionally, autoimmune disease [19].

Selective IgA deficiency is the commonest immunodeficiency with an incidence of 1:400-700. Many affected individuals are asymptomatic, but others, and particularly those who have an associated IgG subclass or specific antibody deficiency, suffer from recurrent sinopulmonary infections [20]. In another important antibody deficiency, X-linked agammaglobulinemia, pyogenic infections such as pneumonia first seem after 6-12 months. Unless treated with regular intravenous or subcutaneous immunoglobulin therapy and aggressive antibiotic treatment of acute infections, this progresses rapidly to chronic bacterial bronchitis and irreversible lung damage with bronchiectasis [21].

In the past, many cases of childhood bronchiectasis followed acute lower respiratory tract infections with pertussis, measles, or tuberculosis. This is now rare. Virtually all children with normal immune function will make a full recovery from pneumonia or bronchiolitis, even if the acute episode was severe. However, there are important exceptions to this rule. Adenovirus serotypes 3, 4, 7, and 21 can all cause severe bronchiolitis, pneumonia and death. Up to 40-70% of survivors are left with permanent damage to the airways (bronchiolitis obliterans) with segmental or lobar atelectasis, areas of hyperinflation and impaired lung function. A quarter of children with bronchiolitis obliterans subsequently develop bronchiectasis [22]. Typically, these children are left with persistent wheeze (which responds poorly to bronchodilators), a persistent cough (which is initially dry but then becomes productive of purulent sputum), and the characteristic radiologic changes of bronchiolitis obliterans. Swyer-James or MacLoed's syndrome, where there is a small hyperlucent lobe with impaired perfusion and ventilation, has also been described after these infections. Similar damage can follow severe mycoplasmal pneumonia. Coinfection with both adenovirus and Mycoplasma pneumoniae is particularly devastating [23].

Primary ciliary dyskinesia (PCD) may present in newborn infants with tachypnea or pneumonia, sometimes associated with nasal obstruction and a mucopurulent discharge. In the older infant, it typically presents with a persistent productive cough, atypical asthma, or occasionally severe gastro-esophageal reflux, and later with the features of bronchiectasis. Half of children with classic Kartagener syndrome have situs inversus and dextrocardia in addition to PCD and there is an increased incidence of congenital heart disease, hydrocephalus and esophageal atresia [24].

## The Role of allergy and asthma

Allergy and asthma are important causes of wheezing and probably generate the most inquires by the parents of a wheezing infant. Three identified patterns of infant wheezing are*: the transient early wheezer*, 19.9% of the general population, had wheezing at least once with a lower respiratory infection before the age of 3 years but never wheezed again; *the persistent wheezer*, 13.7% of the general population, had wheezing episodes before 3 years and was still wheezing at 6 years; *the late onset wheezer*, 15% of the general population, had no wheezing by 3 years, but will wheeze by 6 years. The other half of children would never wheeze by 6 years. Of all the infants who wheeze before 3 years old, almost 60% stop wheezing by 6 years. Risk factors for persistent wheezing include maternal asthma, maternal smoking, persistent rhinitis (apart from acute upper respiratory tract infections), and eczema at < 1 year of age [25].

**Figure 1:**
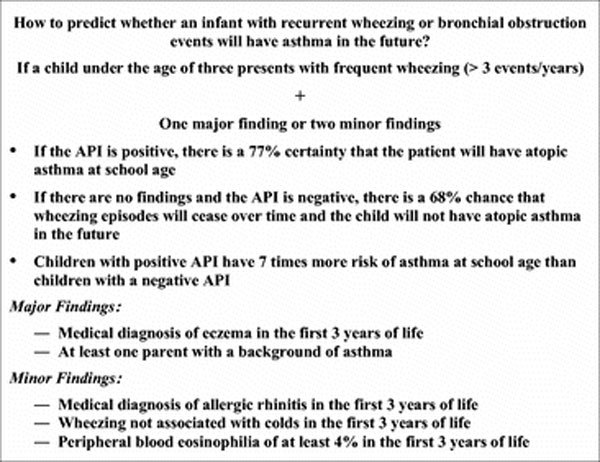
**Assessing the risk of asthma in infants and school children**.

The asthma predictive index (API) was developed 10 years ago by using data from 1246 children in the Tucson Children's Respiratory Study birth cohort. It was based on factors that were found during the first 3 years of life to predict continued wheezing at school age [26]. A loose index (< 3 episodes/year and one of the major or 2 of the minor criteria) and a stringent index (> 3 episodes/year and one of the major or 2 of the minor criteria) were created (Figure 1). The most impressive aspect of the API is its ability to rule out the likelihood of asthma by school age in young children with wheezing [27]. For children who are early wheezers during the first 3 years of life, API negative predictive values ranged from 93.9% at 6 years of age to 86.5% at 13 years of age. For children who are early frequent wheezers during the first 3 years of life, the negative predictive values were 91.6 and 84.2% for 6 and 13 years of age, respectively [28].

## Anatomic disorders

Congenital malformations of the respiratory tract cause wheezing in early infancy. These findings can be diffuse or focal and can be from an external compression or an intrinsic abnormality. External vascular compression includes a vascular ring, in which the trachea and esophagus are surrounded completely by vascular structures, or a vascular sling, in which the trachea and esophagus are not completely encircled [30].

Laryngomalacia patients typically have high-pitch inspiatory stridor that increases with crying and when supine. It rarely interferes with feeding. Symptoms are usually present at birth or within the first 4-6 weeks of life and resolves by its own within 18-24 months. Other anomalies include double aortic arch, innominate artery compression, aberrant right subclavian vein, and pulmonary artery sling. Bronchogenic cysts can also cause extrinsic compression of the trachea. Tracheomalacia may be observed in association with laryngomalacia or as an isolated lesion. Tracheal stenosis can be a complication of long-term endotracheal intubation or a congenital lesion. Complete tracheal rings are a subset of tracheal stenosis with severe biphasic airway sounds [31].

Recurrent or persistent chest infections are common in children with congenital abnormalities of the airways, lung parenchyma, and pulmonary vasculature. For example, repeated episodes of pneumonia are often the presenting feature of lobar sequestration, bronchial stenosis and bronchomalacia, and cystic adenomatoid malformations of the lung. Such an abnormality should be suspected if one lobe is repeatedly infected or if there is incomplete resolution after treatment [32].

Children born with esophageal atresia and tracheaesophageal fistula often have repeated episodes of pneumonia and bronchitis in early life as a result of persisting abnormalities of airway and esophageal function. The condition has an incidence of 1:3000 and associated vertebral, anal, cardiac, renal, or limb defect are seen in up to 50% of affected children. It can cause wheezing with or without direct aspiration into the tracheobronchial tree. Without aspiration, the reflux is thought to trigger a vagal or neural reflex, causing increased airway resistance and airway reactivity [33].

Cardiovascular causes of wheezing may be related to dilated chambers of the heart including massive cardiomegaly, left atrial enlargement, and dilated pulmonary arteries. Pulmonary edema caused by heart failure can also cause wheezing by lymphatic and bronchial vessel engorgement that leads to obstruction and edema of the bronchioles and further obstruction [34].

Foreign body aspiration can cause acute or chronic wheezing. It is estimated that 78% of those who die of foreign body aspiration are between 2 months and 4 years old. Even in young infants, a foreign body can be ingested if given to the infant by another person such as an older sibling. Infants who have atypical histories or misleading clinical and radio-logic findings may be misdiagnosed with asthma or another obstructive disorder as inflammation and granulation develop around the foreign body. Esophageal foreign body can transmit pressure to the membranous trachea, causing compromise of the airway lumen [35].

Trauma and tumors are much more rare causes of wheezing in infants. Trauma of any type to the tracheobronchial tree can cause an obstruction to airflow. Accidental or nonaccidental aspirations, burns, or scalds of the tracheobronchial tree can cause inflammation of the airways and subsequent wheezing [33].

## Clinical manifestations and differential diagnosis

Detailed history laying stress on family history of cystic fibrosis, immune deficiencies, asthma in a 1st degree relative, or any other recurrent respiratory condition in children should be obtained. Social history should include an environmental history including any smokers at home, number of sibling, occupation of inhabitants of the home, pets, tuberculosis exposure, and concerns regarding home environment (eg, dust mites, construction dust, heating and cooling techniques, mold, cockroaches) [36].

On physical examination, evaluation of the patient's vital signs with special attention to the respiratory rate and the pulse oximetry reading for oxygen saturation is an important initial step. There should also be a thorough review of the patient's growth chart for signs of failure to thrive. Biphasic wheezing can occur if there is a central, large airway obstruction. The lack of audible wheezing is not reassuring if the infant shows other signs of respiratory distress because complete obstruction to airflow can eliminate the turbulence, which causes the sound to resonate. Aeration should be noted and a trial of a bronchodilator may be warranted to evaluate for any change in wheezing after treatment. Listening to breath sounds over the neck will help differentiate upper air way from lower airway sounds. The absence or presence of stridor should be noted and appreciated on inspiration. It is also useful to evaluate the skin of the patient for eczema and any significant hemangiomas [30]. Differential diagnosis of wheezing according to age is listed in Table 2.

**Table 2:** Causes of Wheezing According to Age

Disease Prevalence	Neonate/Infant	School Age/Adolescent
Common	Bronchiolitis	Asthma
	Asthma	
Less Common	Pulmonary aspiration	Foreign body aspiration
	Gastroesophageal reflux	Anaphylaxis
	Swallowing dysfunction	Atypical pneumonia
	Foreign body aspiration Bronchopulmonary dysplasia	
	Cystic fibrosis	
Uncommon	Congenital heart disease	Defective host defenses
	Defective host defenses	Mediastinal tumors
	Immunodeficiency	Enlarged mediastinal
	Immotile cilia syndrome	lymph nodes
	Congenital structural	Parasitic infestation
	anomalies	Pulmonary
	Tracheobronchomalacia	hemosiderosis
	Vascular ring	*α*_1_- antitrypsin
	Lobar emphysema	deficiency
	Cystic abnormalities	
	Tracheoesophageal fistula	

Quoted from Shah (2004) [10].

## Investigations

Most infants with a first wheezing (even those with recurrent wheezing) do not need any investigations. The plain chest x-ray is valuable in assessing the severity and distribution of lung involvement. Wide-spread changes such as bronchial wall thickening or inflammation involving several lobes suggest a systemic disorder such as cystic fibrosis (CF), ciliary dyskinesia, or an immunodeficiency disorder. Focal changes are more common if there is congenital abnormality, an inhaled foreign body or bronchial obstruction for some other reason. High resolution computerized tomography (HRCT) is more sensitive than plain radiographs at revealing bronchiectasis: it has largely replaced bronchography. It can also show localized areas of gas-trapping (hyperinflation) and interstitial fibrosis not evident on the chest x-ray, for example in children with bronchiolitis obliterans. CT scanning and magnetic resonance imaging are both helpful for assessing congenial anatomic abnormalities, such as sequestration or cystic adenomatoid malformations. Isotope scans provide useful evidence about regional ventilation and perfusion. All children with persistent cough should have their sweat electrolytes measured. The sweat test remains the standard diagnostic test for CF, although CF gene mutation studies are being used increasingly. However, the necessity for CF screening is race-dependent. Other investigations include bacteriological studies on sputum if this can be produced; viral and mycoplasmal antibody levels; tuberculin skin testing; and in selected cases, immune function tests. All children should have a full blood count and white cell differential: persistent lymphopenia or neutropenia may be present even when the child is well [37].

## Treatment

Treatment of an infant with wheezing depends on the underlying etiology. Response to bronchodilators is unpredictable, regardless of cause, but suggests a component of bronchial hyperreactivity. It is appropriate to administer albuterol aerosol and objectively observe the response. For infants, it is acceptable to continue to administer inhaled medications with mask and spacer if a therapeutic benefit is demonstrated [38]. The use of ipratropium bromide is also useful in infants with significant tracheal and bronchomalacia who may be made worse by *β*-2 agonists such as albuterol because of the subsequent decrease in smooth muscle tone [25].

A trial of inhaled steroids may be warranted in a patient who has responded to multiple courses of oral steroids, has moderate to severe wheezing, or a significant history of atopy including food allergy or eczema. Inhaled steroids are appropriate for maintenance therapy in patients with known reactive airways but are controversial when used for episodic or acute illnesses [39].

Infants with acute bronchiolitis who are experiencing respiratory distress should be hospitalized; the mainstay of treatment is supportive. If hypoxemic, the child should receive cool humidified oxygen. Sedatives are to be avoided because they may depress respiratory drive [8]. Corticosteroids, whether parenteral, oral, or inhaled, have been used for bronchiolitis despite conflicting and often negative studies. Differences of diagnostic criteria, measures of effect, timing and route of administration, and severity of illness complicate these studies. Corticosteroids are not recommended in previously healthy infants with RSV. Ribavirin, an antiviral agent administered by aerosol, has been used for RSV bronchiolitis in infants with congenital heart disease or chronic lung disease [40].

Reduction in the severity and incidence of bronchiolitis because of RSV is possible through the administration of pooled hyper-immune RSV intravenous immunoglobulin (RSV-IVIG), and intramuscular monoclonal antibody to the RVS F protein, before and during RSV season. Palivizumab is recommended for infants < 2 years of age with chronic lung disease (bronchopulmonary dysplasia) or prematurity. Meticulous hand washing is the best measure to prevent nosocomial transmission [12].

A practical approach for preschool wheezers was recently proposed. In a preschooler with recurrent wheezing, the possibility of an underlying allergy should be assessed (by skin prick testing or determination of specific IgE). If allergy is not present, the wheezing should be considered as nonallergic (viral-induced wheezing), and usually little maintenance treatment is needed, as most of the children will grow out of it. Therefore, treatment should be focused on treating the symptoms (with beta-agonists, short coursed of prednisolone, etc). If the symptoms are frequent a leukotrienereceptor antagonist might be considered in the first place, before starting ICS. In contrast, if allergy is present (positive skin prick test or positive specific IgE) there is an increased risk that the child will continue wheezing beyond preschool age. In this case ICS might be necessary to control the underlying inflammation and to prevent symptoms [41].

## Conclusion

Wheezing in infants could be a sign of diverse clinical conditions. Meticulous search for the cause and evaluation of the clinical status are mandatory before conducting therapy. Not every wheeze is indicative of asthma but prediction of asthma in persistent wheezers is possible. Testing for allergy in these infants is worthwhile and can be of significant value in avoidable allergens. Morbidity may also be diminished and quality of life improved if controller therapy is started in proper time.
